# Cannabinoids for Dermatological Applications: Mechanistic Insights, Clinical Evidence, and Emerging Nanotechnology-Enabled Delivery Strategies

**DOI:** 10.3390/pharmaceutics18040469

**Published:** 2026-04-12

**Authors:** Ashutosh Pareek, Lipika Kumari, Lance R. McMahon, Anil Chuturgoon, Aaushi Pareek

**Affiliations:** 1 Department of Pharmacy, Banasthali Vidyapith, Banasthali 304022, India; aaushipareek@banasthali.in; 2 Department of Bioscience and Biotechnology, Banasthali Vidyapith, Banasthali 304022, India; 3 Department of Pharmaceutical Sciences, Jerry H. Hodge School of Pharmacy, Texas Tech University Health Sciences Center, Amarillo, TX 79106, USA; 4 Discipline of Medical Biochemistry, School of Medicine, University of KwaZulu-Natal, Durban 4041, South Africa

**Keywords:** cannabidiol, dermatology, psoriasis, atopic dermatitis, skin cancer, nanotechnology

## Abstract

Cannabinoids (CBs) derived from *Cannabis sativa* have attracted growing interest for dermatological applications due to their anti-inflammatory, antiproliferative, antimicrobial, antifibrotic, and antipruritic properties. However, their clinical translation is significantly limited by physicochemical and pharmacokinetic challenges, including poor aqueous solubility, lipophilicity, instability, variable skin penetration, and inconsistent bioavailability. At the molecular level, CBs modulate keratinocyte proliferation, sebocyte activity, fibroblast function, melanocyte balance, and immune signalling through CB1/CB2 receptors, TRP channels, and PPARγ pathways. Evidence supports their potential in the treatment of psoriasis, atopic dermatitis, acne, allergic contact dermatitis, pruritus, scleroderma, and skin cancers. Clinical evidence remains preliminary: topical and oral formulations have demonstrated anti-inflammatory, antiproliferative, antibacterial, and antifibrotic effects, with improvements in pruritus, lesion severity, and quality of life in early-phase studies. However, most trials are small, uncontrolled, and lack placebo comparators, limiting generalisability. To overcome formulation barriers and enhance dermal delivery, advanced pharmaceutical strategies such as liposomes, nanoemulsions, polymeric nanoparticles, micelles, and transdermal systems have been investigated to improve stability, controlled release, and targeted skin deposition while minimising systemic exposure. This review integrates mechanistic insights, clinical evidence, and emerging nanotechnology-enabled delivery approaches, emphasising rational formulation design and translational considerations necessary for advancing CBs toward standardised and clinically reliable dermatological therapeutics.

## 1. Introduction

Increasing interest in plant-derived bioactives reflects growing emphasis on sustainability and consumer preference for naturally sourced dermatologic ingredients [1]. *Cannabis sativa* (*C. sativa*), one of the oldest medicinal plants, has recently attracted significant attention owing to widespread legalisation in several countries and its diverse chemical profile [2]. This has contributed to the rapid expansion of cannabis-derived products in dermatology. However, uncertainty persists regarding their therapeutic advantages, largely due to variability in product composition and limited standardisation. Furthermore, the translation of cannabinoids into standardised dermatological therapeutics is complicated by physicochemical challenges, including poor aqueous solubility, high lipophilicity, chemical instability, and variable skin penetration, all of which may compromise formulation consistency and reproducible clinical outcomes [3].

Recent research indicates the presence of more than 1200 distinct compounds in cannabis, including over 100 cannabinoids (CBs) belonging to the C21 or C22 molecular group [4]. In the past two decades, interest in the therapeutic use of CBs has grown considerably, with multiple clinical trials investigating their potential applications in pain, epilepsy, neurological disorders, obesity, and cancer [5]. Cannabis has been legalised in some countries, and scholarly publications related to the chemistry and pharmacology of cannabinoids have increased markedly since 2013; however, research in dermatology remains at an early stage, and additional work is required to establish comprehensive knowledge of CBs’ effects on the human body [6].

CBs are broadly classified into phytocannabinoids, endocannabinoids, and synthetic cannabinoids, each of which interacts with multiple molecular targets [7,8]. Evidence suggests that CBs regulate keratinocyte proliferation [9], fibroblast activity [10], sebocyte function [11], melanocyte balance, and immune responses, thereby influencing skin homeostasis and inflammation [8,12,13]. This has prompted growing interest in their possible therapeutic value for conditions such as psoriasis [14], atopic dermatitis [15], acne [16], allergic contact dermatitis [17], pruritus [18], systemic sclerosis [19], and skin cancers [8,13,20].

Accordingly, this review integrates mechanistic insights, emerging clinical evidence, and advanced formulation approaches aimed at overcoming delivery barriers, with particular emphasis on nanotechnology-enabled strategies that may facilitate the translation of cannabinoids into standardised and clinically reliable dermatological therapeutics.

## 2. Methodology

This review was conducted as a narrative synthesis of the published literature. Relevant studies were identified through searches in PubMed, Scopus, and Web of Science using combinations of the terms “cannabinoids”, “endocannabinoid system”, “skin diseases”, “psoriasis”, “atopic dermatitis”, “acne”, “pruritus”, and “dermatology.”

The initial search yielded approximately 423 records, from which duplicates were removed. Titles and abstracts were screened to exclude irrelevant studies, resulting in 237 articles for full-text assessment. Studies were included if they addressed mechanistic insights, formulation strategies, or therapeutic applications of cannabinoids in dermatology. Exclusion criteria comprised non-English publications, studies lacking methodological clarity, and reports not directly related to dermatological outcomes.

Additional relevant articles were identified through manual screening of reference lists. Following full-text evaluation, a subset of the most relevant studies was selected for thematic synthesis, while additional supporting literature was incorporated to provide comprehensive background and contextual understanding.

## 3. Types of Cannabinoids

**Figure 1:**
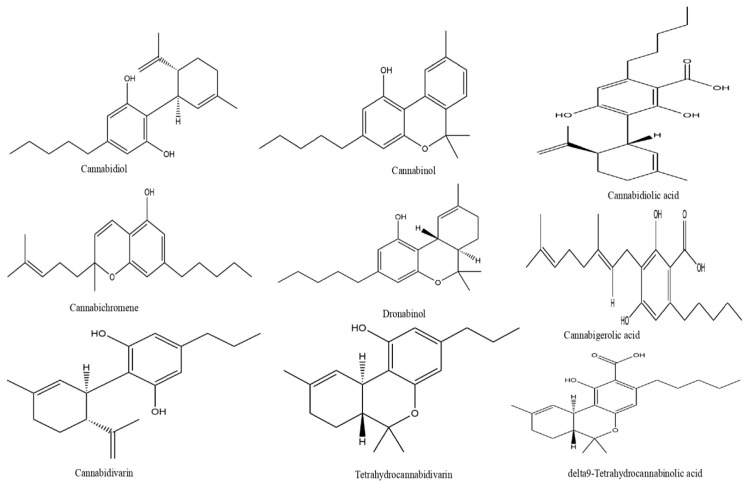
Chemical structures of selected important cannabinoids.

CBs can be classified into three broad categories depending on their origin. Endocannabinoids (ECBs) are compounds naturally produced in the human body. Kendall et al. [7] identified nine endocannabinoids in human epidermis: anandamide (AEA), 2-arachidonoylglycerol (2-AG), N-palmitoylethanolamine (PEA), N-oleoylethanolamine (OEA), N-linoleoylethanolamine (LEA), N-stearoylethanolamine (SEA), N-α-linolenoylethanolamine (ALEA), N-eicosapentaenoylethanolamine (EPEA), and N-docosahexaenoylethanolamine (DHEA). Phytocannabinoids (PCBs) derive from plants, primarily *C. sativa* trichomes. Synthetic cannabinoids (SCs) like dronabinol and nabilone are chemically produced [5,8]. The resin-producing trichomes of *C. sativa* contain an abundance of PCBs. One of the PCBs, delta-9-tetrahydrocannabinol (THC-9), is classified as a psychoactive compound, whereas most other PCBs, such as cannabidiol (CBD) and cannabigerol (CBG), are not psychoactive (Figure 1). *C. sativa* includes different varieties; regulatory agencies usually categorise these into two main phytochemical classes [8]. Hemp has a low quantity of THC and high CBD levels, whereas marijuana has high THC levels (typically over 0.3% by dry weight) [21].

Hemp seeds are abundant in beneficial omega-3 fatty acids and protein (25–35%) yet contain few PCBs and do not have terpenoids [22]. Essential oils, including terpenoids like α-pinene, myrcene, and β-caryophyllene, are typically extracted from hemp flowering tops and leaves using steam distillation [23]. However, the steam distillation process does not yield substantial amounts of PCBs in the volatile fraction [24]. CBD may be present in significant quantities in ethanolic and supercritical CO_2_ extractions from the whole hemp plant or from its flowering tops and leaves [25]. These extracts can often be called by a number of names, including PCB-rich hemp oil or extract, full-spectrum hemp extract, and broad-spectrum hemp extract.

CBs produce various pharmacological effects, including anticonvulsant, antispasmodic, pain-relieving, anti-nausea, neuroprotective, and anti-inflammatory effects [26,27]. Consequently, they have been widely used in treating various clinical conditions, with chronic pain among the most common applications. They may be appropriate replacements or supplements for people with illnesses such as acid reflux disease, ulcers, kidney issues, and other disorders where non-steroidal anti-inflammatory medications and opioids are poorly tolerated [28,29]. There is increasing clinical and preclinical evidence suggesting that CBs products could aid in the effort to address the opioid overdose epidemic by helping to prevent drug dependence or the need for escalating doses, which are significant issues associated with opioid use [30,31]. Furthermore, multiple studies have proved that CBs products are useful in controlling conditions such as anorexia [26], chemotherapy side effects [32], migraine [33], sclerosis [34], schizophrenia [33], spasms in the muscles [26], and epilepsy [35]. Moreover, they have been discovered to possess antineoplastic properties [36], showing promise for addressing a range of skin disorders, including skin cancer [8], acne [16], hidradenitis suppurative [37], systemic sclerosis [19], pruritus [18], atopic dermatitis (AD) [15], asteatotic dermatitis [38], allergic contact dermatitis (ACD) [17], and psoriasis [14]. Additionally, they have been examined for their potential application as an ocular hypotensive drug in glaucoma [39].

## 4. Applications in Dermatology

In addition to high levels of expression in the immune and central nervous systems, CB1 and CB2 receptors are also present in the skin [40,41]. In addition, Transient Receptor Potential (TRP) is another type of receptor associated with CBs in the skin [42]. Cellular functions like differentiation, apoptosis, proliferation, and cytokine activity are all regulated by CB [37,40]. CBs mediate the balance of melanocytes, keratinocytes, and sebocytes via both CB1R/CB2R-dependent and independent mechanisms [12,13,43]. Through the p38 MAP kinase pathway, CB1R activity in the stratum spinosum and stratum granulosum and CB2R activity in the basal layer of the epidermis can enhance the methylation of human keratinocyte DNA, hence limiting the growth of keratinocytes [44]. However, independent of CB1R/CB2R involvement, ECBs prevent keratinocyte proliferation. This is probably caused by the activation of GPR55, a G-protein-coupled receptor, or peroxisome proliferator-activated receptor gamma (*PPAR-γ*) [9].

The interactions of CBs with cellular functions in skin offer a justification for the possible application of CBs in diverse dermatological issues such as acne vulgaris [16,45], ACD [17,37], asteatotic dermatitis [10,38], AD [10,46], psoriasis [43,47], Kaposi Sarcoma [37], pruritus [37,38,43], skin cancer [8,13,37,43] and systemic sclerosis cutaneous manifestations [37]. According to studies investigating the anti-acne properties of CBs, a “three-fold cellular anti-acne effect” has been reported, which involves (a) reversing increased lipid levels triggered by factors promoting acne, (b) inhibiting the proliferation of sebocytes, and (c) blocking the activation of Toll-like receptors responsible for increasing pro-inflammatory cytokines [42]. CBs possess antibacterial properties [9] and may have potential efficacy against *Propionibacterium acnes*, the bacterium known to contribute to acne [48]. Given the skin’s reliance on the endocannabinoid system (ECS) for regulation, topical CB application and effective skin absorption could be beneficial for various skin conditions or overall skin health [8]. Applying CBs topically avoids first-pass metabolism [49]. Nevertheless, integrating CBs into topical formulations poses challenges because these compounds are highly lipophilic, resulting in poor water solubility [50], limited skin penetration [34], and susceptibility to degradation by factors such as light [51], temperature [52], and autoxidation [53]. These challenges highlight the potential of CBs for integration into sophisticated drug delivery systems, like nanoparticles, liposomes, and micelles, for administration either topically or via other routes [33].

## 5. Dermatological Indications

**Figure 2:**
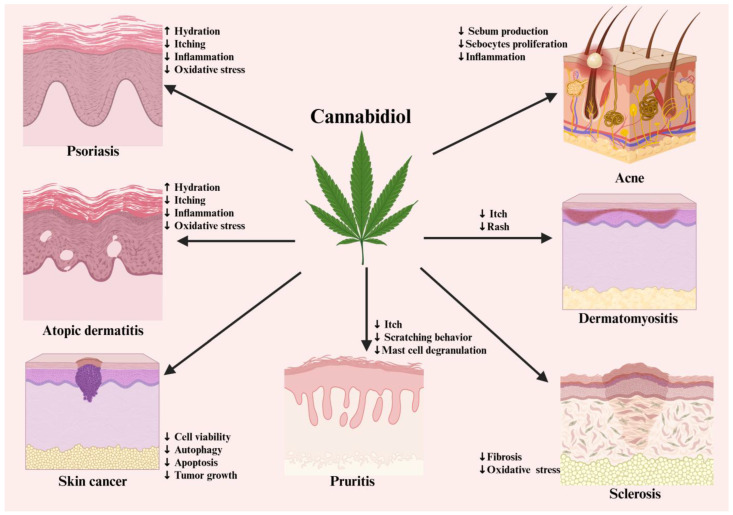
A schematic diagram of cannabinoids and their potential role in the treatment of various skin diseases, which include Psoriasis, Atopic dermatitis, Skin Cancer, Acne, Dermatomyositis, Sclerosis and Pruritus. Created in BioRender. Pareek, A. (2026) https://BioRender.com/anqkrcu.

This section provides an overview of studies investigating the impact of CBs and the influence of ECS in different inflammatory skin disorders (Figure 2).

### 5.1. Psoriasis

Approximately 2% to 3% of people worldwide suffer from psoriasis, which can cause significant morbidity and often intensifies depression and anxiety in patients [54]. The epidermal growth system regulates the growth and formation of new blood vessels in skin cells, thereby managing their development and eventual apoptosis [40].

**Figure 3:**
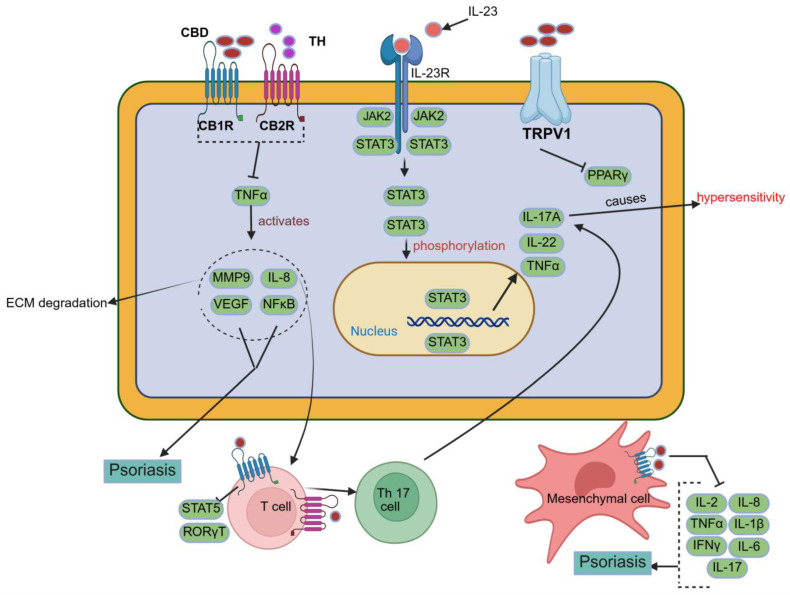
Cannabinoids’ mechanism of action in Psoriasis: CBD inhibits *NFκB* transcription, which is triggered by *TNF-α*, a major driver of inflammation in psoriasis. This is achieved through the activation of PPAR-γ, a receptor that regulates cell growth and inflammation. Deficiency in CB2R impairs CD4^+^ T cell differentiation by affecting the *STAT5*/*RORγT* signalling pathway. Human mesenchymal stem cells express all components of ECS, including CB1R and CB2R. Stimulation of CB2R in these cells reduces the pro-inflammatory cytokines. The *IL-23* receptor, crucial in psoriasis, activates *STAT3* phosphorylation through *JAK2* and *TYK2* signalling. CBD inhibits *JAK2* and *STAT3*, reducing cytokines responsible for Psoriasis, such as *IL-17A*, *IL-22*, and *TNF-α*. Arrows indicate flow & regulation, the dotted lines represent non-significant correlations, and the T-shaped symbols denote inhibition. CBD, Cannabidiol; *TNF,* Tumour necrosis factor; *NFκB,* Nuclear factor kappa-light-chain-enhancer of activated B cells; PPAR-γ, Peroxisome proliferator-activated receptor gamma; CB2R, Cannabinoid receptor 2; CD4^+^, Cluster of differentiation 4; *STAT,* Signal transducer and activator of transcription; *RORγ,* Retinoic acid-related orphan receptor gamma; ECS, Endocannabinoid system; CB1R, Cannabinoid receptor 1; IL, Interleukin; *JAK2,* Janus kinase 2; *TYK2,* Tyrosine-protein kinase. Created in BioRender. Pareek, A. (2026) https://BioRender.com/19m0n2i.

CBD has shown potential therapeutic relevance in psoriasis due to its anti-inflammatory and antiproliferative properties. Evidence suggests that CBD may modulate keratinocyte activity and reduce the release of pro-inflammatory cytokines, like *TNF-α*, *IL-2*, and *IFN-γ*, which play key roles in developing the condition, as shown in Figure 3 [55,56].

According to Derakhshan and Kazemi [57], CBD has a therapeutic role in psoriasis by exerting antiproliferative effects on keratinocytes and anti-inflammatory effects, which include stimulating the vagal nerve, which releases acetylcholine, and modulating the immune system by preventing cytokine-producing macrophages from producing *TNF-α* [37]. Psoriatic skin has been shown to exhibit marked overexpression of nuclear receptor-interacting protein 1 (*NRIP1*), a transcriptional coregulator implicated in epidermal hyperproliferation and inflammatory signalling. In a mechanistic study, Luan et al. demonstrated that *NRIP1* overexpression in psoriatic lesions promotes keratinocyte proliferation, while targeted downregulation of *NRIP1* in HaCaT cells significantly reduced cell proliferation, IL-17 secretion, and NF-κB activity. These findings identify *NRIP1* as a pathogenic mediator and a potential multimodal therapeutic target in psoriasis. Although direct modulation of *NRIP1* by cannabidiol has not been experimentally established, the antiproliferative and anti-inflammatory actions of cannabinoids suggest that *NRIP1*-associated pathways may represent a relevant downstream axis through which cannabinoids could exert therapeutic effects in psoriatic disease [58].

Norooznezhad and Norooznezhad [59] proposed the utilisation of the synthetic cannabinoid JWH-133 to target angiogenesis, a process implicated in the pathogenesis of psoriasis. This compound exhibits antiangiogenic and anti-inflammatory properties, as it significantly suppresses the release of angiogenic growth factors, cytokines, and MMPs, including *IL-8*, *IL-17*, *VEGF*, *bFGF*, and *HIF-1α*. Consequently, JWH-133 can target inflammation and angiogenesis, as these are important factors in developing psoriasis.

These findings suggest that CBs may be beneficial in psoriasis by hindering keratinocyte proliferation, mitigating inflammation, and modulating angiogenesis. These findings suggest that CBs may be beneficial in psoriasis by hindering keratinocyte proliferation, mitigating inflammation, and modulating angiogenesis. However, most of the evidence derives from preclinical models or small exploratory studies, and robust randomised controlled trials are still lacking [2].

### 5.2. Atopic Dermatitis

**Figure 4:**
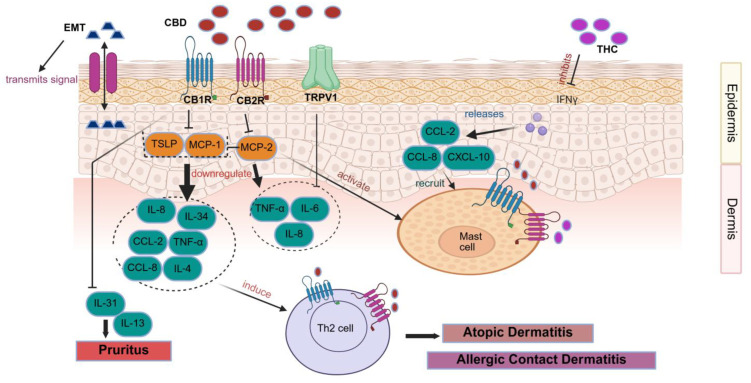
Cannabinoids reduce inflammation by suppressing (denoted by T-shape symbol) pro-inflammatory cytokines such as *TNF-α*, *IL-6*, and *IL-8*. Increased *TSLP*, *IFN-γ*, *CCL8*, and *IL-4* levels in CB1R-deficient mice drive Th2-type inflammation. CBD activates CB1R and CB2R to decrease mast cell recruitment-related cytokines, including *MCP-1* and *MCP-2*. The development and activity of mast cells, which express CB1R and CB2R, are reduced by CBD. THC limits myeloid immune cell infiltration into afflicted tissues by lowering pro-inflammatory mediators (*CCL2*, *CCL8*, and *CXCL10*) derived from keratinocytes that *IFN-γ* triggers. CB1R/CB2R agonists reduce persistent itching by inhibiting *IL-13* and *IL-31*, two important cytokines associated with itching. CBD, Cannabidiol; IL, Interleukin; *TNF*, Tumour necrosis factor; CB1R, cannabidiol 1 receptor; *TSLP*, thymic stromal lymphopoietin; *IFN*, interferon; *CCL*, Chemokine (C-C motifs) ligand; CB2R, cannabidiol 1 receptor; *MCP*, monocyte chemoattractant protein; THC, tetrahydrocannabinol; *CXCL*, chemokine (C-X-C motif) ligand. Created in BioRender. Pareek, A. (2026) https://BioRender.com/gr1cs6n.

Numerous studies have demonstrated a strong link between CBD and AD (Figure 4) [60], with treatments targeting the ECS gaining increasing attention due to their relevance in the disease’s pathophysiology [60]. Proinflammatory cytokines, including *IL-4*, *IL-13*, and *IL-31*, are overproduced in AD due to abnormal *JAK/STAT* pathway activation, and these cytokines are directly associated with inflammation, itching, and barrier failure [61,62]. Conventional AD therapies typically rely on anti-inflammatory agents such as topical steroids and calcineurin inhibitors, combined with moisturisers to restore the skin barrier and reduce microbial colonisation. However, growing evidence suggests that targeting CB1R within the skin’s ECS may represent a promising therapeutic strategy [63]. In vivo investigations revealed that eosinophil activity and mRNA expression of *IL-4*, *TSLP*, and *CCL8* were significantly elevated in inflamed tissues, while keratinocytes lacking CB1R secreted higher levels of *TSLP* under both normal and Th2-driven inflammatory conditions, thereby exacerbating allergic inflammation. These findings underscore the critical role of keratinocyte CB1R in maintaining epidermal barrier stability and limiting Th2-mediated immune responses [64]. Additionally, studies have shown that CB1R agonists dose-dependently reduced mast cell proliferation and suppressed antigen-dependent, IgE-mediated release of inflammatory mediators from RBL-2H3 cells without causing cytotoxicity. Topical administration of CB1R agonists further decreased circulating histamine levels and mast cell migration into the skin [65]. Enhancing CB1R activity in keratinocytes has also been shown to improve skin barrier function, reduce Th2-type inflammation, and inhibit mast cell activity; notably, α-OOS alleviated oxazolone-induced AD symptoms by reducing trans-epidermal water loss, decreasing skin thickness, lowering surface pH, and increasing hydration [66]. These findings collectively indicate that topical formulations containing ECS receptor agonists or degradation inhibitors hold significant therapeutic potential for AD management [67]. Topical administration of CBG substantially reduced skin inflammation and enhanced epidermal barrier function, which involved a mouse model of AD [68]. Supporting this, several clinical studies have confirmed the efficacy of topical CBD in reducing AD symptoms. A recent controlled trial using a combined CBD and CBD-acid oil in dogs with AD demonstrated a reduction in pruritus, though no significant improvements were noted in serum inflammatory markers or skin lesions [69]. Innovative delivery systems are also under exploration, such as a polycaprolactone patch designed for sustained hemp seed oil release. Trials with three human volunteers and skin models showed that up to 55% of the oil was released within 6 h, resulting in a 25% improvement in skin moisturisation, highlighting the potential of controlled-release patches as convenient therapeutic tools [70]. Because it suppresses the Th2 immune response, CBC is a viable therapy option for AD and may help reduce its symptoms [71]. Preclinical and early clinical findings indicate that CBD and CBD-containing formulations may help alleviate hallmark AD symptoms such as itching, irritation, and skin dryness.

### 5.3. Allergic Contact Dermatitis

CBD may hold therapeutic potential in ACD due to its anti-inflammatory properties (Figure 4) [72]. Evidence from animal studies shows that CB1R/CB2R^−^/^−^mice exhibit heightened sensitivity to contact allergens such as 2,4-dinitrofluorobenzene (DNFB), while treatment with THC following DNFB exposure reduced Gr-1 granulocyte infiltration and ear oedema. Similarly, mice lacking FAAH, the enzyme responsible for the degradation of the ECB agonist AEA, displayed diminished allergic responses, suggesting that activation of CB1R and CB2R can alleviate inflammation in ACD. However, an important exception was observed when immediate application of a CB2R antagonist initially reduced inflammation but later exacerbated it, indicating a complex, time-dependent role of CB2R in regulating immune responses [73]. Supporting this, other studies have shown that CB2R activation may exert pro-inflammatory effects. In murine ACD models induced by DNFB, CB2R-deficient mice displayed reduced ear swelling after 24 h, while CB2R activation by ECB agonist 2-AG increased ear weight and upregulated *MCP-1* and *IL-8* expression in human HL-6 cells. These findings suggest a dual and context-dependent role of CB2R in ACD, with both pro- and anti-inflammatory actions reported [74,75]. Given the variability in outcomes, more mechanistic and translational studies are needed before therapeutic implications can be drawn.

### 5.4. Acne and Seborrhoea

ECBs could be a promising treatment for acne and seborrhoea. Since human sebaceous glands have CB1 and CB2 receptors, CBD might affect inflammation in sebocytes and the production of lipids [11]. CBD targets the pathways that lead to inflammation, sebocyte proliferation, and sebum production, indicating the potential of CBD as an anti-inflammatory and lipostatic agent in acne, as demonstrated in several in vitro studies [15,16,43]. The mechanism of action of CBD is not fully established, and its targets likely include receptors aside from CB1 and CB2.

A detailed investigation by Oláh et al. [43] examined the effects of cannabidiol on sebaceous gland function using human SZ95 sebocytes. Exposure to CBD at concentrations of 1–10 μM for 24 h did not significantly alter basal lipid synthesis. However, when sebocytes were pretreated with the endocannabinoid anandamide (AEA), CBD dose-dependently suppressed AEA-induced lipogenesis. This selective inhibition of stimulated, but not basal, lipid production suggests that CBD does not act as a direct agonist of classical endocannabinoid receptors. Instead, the study demonstrated that CBD exerts its sebostatic effects primarily through CB1/CB2-independent mechanisms, notably via activation of the transient receptor potential vanilloid 4 (TRPV4) channel and downstream *MAPK*/*ERK* signalling pathways [43].

In the same study, the antiproliferative effects of CBD were evaluated in SZ95 sebocytes. CBD significantly reduced sebocyte proliferation at concentrations of 1–10 μM without inducing acute cytotoxicity, indicating a cytostatic rather than cytotoxic effect at therapeutically relevant doses. In contrast, prolonged exposure (6 days) or higher concentrations (50 μM) resulted in reduced cell viability, reflecting dose- and time-dependent cytotoxicity. Notably, TRPV4-mediated signalling was implicated in the regulation of sebocyte proliferation and lipid synthesis, whereas CBD’s anti-inflammatory actions were shown to occur independently of TRPV4 activation [43].

Dobrosi and colleagues [76] conducted in vitro experiments on cultured human SZ95 sebocytes and discovered AEA and 2-AG. Further, the cells expressed CB2R but not CB1R. By upregulating genes implicated in this pathway, the ECBs improved lipid synthesis dose-dependently. In addition, apoptosis was the cause of cell death for both AEA and 2-AG. Through selective CB2R-coupled signalling via the *MAPK* pathway, these outcomes were attained. Drugs which can either block CB2R receptors on sebocytes (CB2R antagonists) or reduce the generation of these ECBs in the impacted sebaceous glands (DAGL inhibitors) may be effective in treating acne and seborrhoea.

Jin and Lee [77] conducted an in vitro study to assess the anti-inflammatory, antimicrobial, and anti-lipogenic effects of hexane extracts from hemp seeds on human HaCaT keratinocytes. Studies on *C. acnes*-stimulated HaCaT cells have demonstrated anti-inflammatory and antibacterial properties of the hexane extracts. Key gene expressions in signalling pathways (*NFκB* and *MAPK*), inflammatory cytokines (*IL-8* and *IL-1β*), and enzymes (*iNOS* and *COX-2*). The extracts were also found to inhibit 5-lipoxygenase and *MMP-9* activity, which led to increased collagen production in vitro. Moreover, when applied to *IGF-1*-stimulated lipogenesis, they showed anti-lipogenic and anti-inflammatory properties. Hence, these extracts from hemp seeds could be utilised for acne vulgaris treatment.

In general, the ECB appears to be a promising approach for controlling sebum production and providing therapeutic benefits for conditions like acne and seborrhoea.

### 5.5. Sclerosis

**Figure 5:**
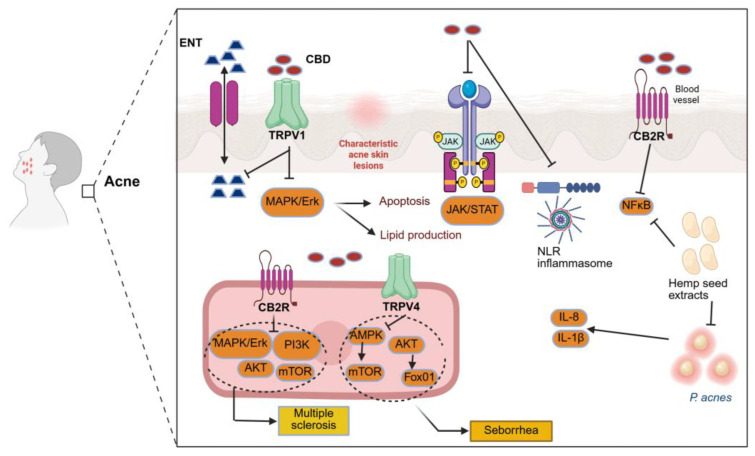
ECBs promote lipid production and lead to the death of sebocytes (oil-producing skin cells) through mechanisms that depend on CB2R. CBD’s lipostatic (oil-reducing) action is mediated through TRPV4 ion channel activation and *MAPK*/*ERK* signalling pathway inhibition. Hemp seed hexane extract suppresses the *NF-κB* and *MAPK* pathways in keratinocytes and controls the *AMPK* and *AKT*/*FoxO1* signalling pathways in sebocytes to control lipid synthesis. CBD has anti-inflammatory effects that are linked to agonism of the *TRPV1* receptor, inhibition of *JAK*/*STAT* signalling pathways, suppression of NLR inflammasome complex activation and inhibition of adenosine uptake via ENT. In multiple sclerosis, the *PI3K*/*Akt*/*mTOR* pathway regulates inflammation, particularly in lymphocytes. Indirect inhibition of the *MAPK* pathway contributes to anti-inflammatory actions via phosphorylation of the *PI3K*/*Akt*/*mTOR* pathway. Arrows indicate flow and mediation, and the T-shaped symbols denote inhibition. ECBs, Endocannabinoids: CB2R, cannabidiol 2 receptor; CBD, cannabidiol; TRPV, transient receptor potential vanilloid; *MAPK*, mitogen-activated protein kinase; *ERK*, extracellular signal-regulated kinase; *NFkB*, nuclear factor-kappa B; *AMPK*, Adenosine monophosphate-activated protein kinase; *FoxO1*, Forkhead; *JAK*/*STAT*, Janus kinase/Signal transducers and activators of transcription; NLR, neutrophil-to-lymphocyte ratio; ENT, equilibrative nucleoside transporters; *PI3K*, phosphoinositide 3-kinases; *mTOR*, mammalian target of rapamycin. Created in BioRender. Pareek, A. (2026) https://BioRender.com/4l56smc.

CBD, through its ability to regulate inflammation, may help prevent fibrosis and limit tissue thickening in sclerosing disorders, as dermal fibroblasts express both CB1R and CB2R (Figure 5) [78]. To investigate the role of CBD in fibrosis, researchers induced skin and lung fibrosis in vivo using hypochlorite injections to model diffuse systemic sclerosis [79]. Treatment with WIN 55,212-2, a non-selective cannabinoid agonist, and JWH-133, a selective CB2R agonist, significantly reduced fibrosis in both tissues, decreased fibroblast proliferation, and lowered autoantibody formation, including anti-DNA topoisomerase I. Similar antifibrotic effects of WIN 55,212-2 were later confirmed in a bleomycin-induced systemic sclerosis model [80]. Consistently, CB2 mutant mice were shown to be more susceptible to bleomycin-induced skin fibrosis [81]. Studies on VCE-004.8, a synthetic cannabinoid similar to CBD, demonstrated reductions in vascular collagen deposition, macrophage infiltration, fibroblast growth, and autoantibody production in vivo [82]. Pro-fibrotic proteins like *IL-4* and *IL-13*, which are produced by Th2 cells, encourage fibroblasts to produce collagen, thereby accelerating the fibrotic process. Targeting Th2 cell differentiation is therefore a possible treatment approach [83]. Results from a study pointed to a possible pathophysiological involvement for S100A11 in the lung and skin fibrosis linked to sclerosis, indicating the need for more research into the protein’s functional roles in the development of this disease [84]. While these findings suggest CB2R involvement in modulating fibrosis, they remain experimental, and further evidence is required, including human research.

### 5.6. Dermatomyositis

Dermatomyositis is an idiopathic inflammatory muscle illness. Symptoms include dysphagia, or trouble swallowing; weariness; calcinosis; an altered voice (dysphonia); and dyspnoea, or difficulty breathing due to weakening of the oesophagus and respiratory muscles [85].

Lenabasum, a CB2R agonist, prevented the modulation of cytokines, including *TNF-α*, *IFN-α,* and *IFN-β,* in PBMCs from patients with dermatomyositis [86]. Additionally, lenabasum decreased skin lesion-associated levels of *IL-31*, a cytokine that is more common among people with itchy dermatomyositis. Furthermore, when triggered by a Toll-like receptor 9 (*TLR9*) agonist, CpG oligodeoxynucleotide, it decreased the production of *IL-31* in the fluid from PBMCs obtained from these patients [87].

**Figure 6:**
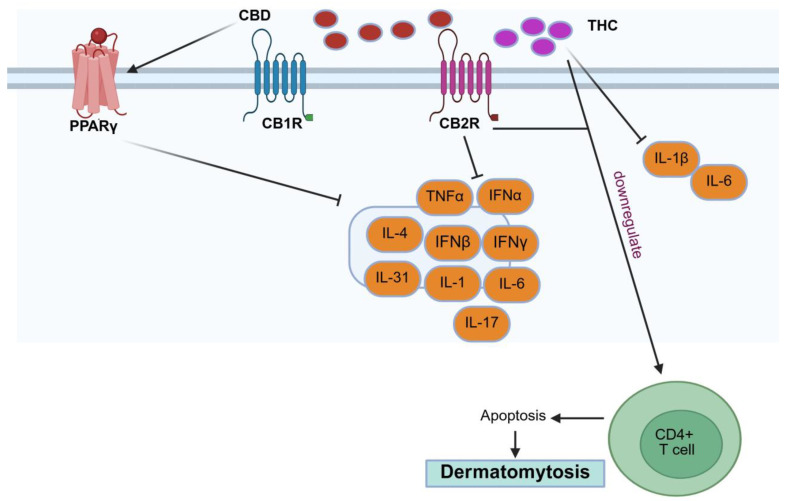
CD4^+^ T cells and pro-inflammatory cytokines, including *IL-31*, *IFN-γ*, and *IFN-β*, are downregulated in response to cannabinoids. *IFN-γ*, *IFN-β*, *IL-31*, and *IL-4* comprise the cytokines produced by skin CB2R cells. It has been demonstrated that CB2R activation inhibits these pro-inflammatory cytokines. CB2R activation reduces pro-inflammatory cytokines linked to DM, such as type I *IFN*, *IL-1*, *IL-6*, *IL-17*, and *TNF-α*. CBD induces transcriptional gene alterations linked to inflammation and cell differentiation by activating the nuclear hormone receptor, PPARγ. Either direct binding to PPARγ or downstream CB2R activation may produce this anti-inflammatory action. CBD suppresses inflammatory cytokine production and regulates immune responses through CB2R activation and PPARγ modulation, representing a novel therapeutic approach for managing dermatomyositis. Arrows indicate flow and mediation, and the T-shaped symbols denote inhibition. CD4^+^, cluster of differentiation 4; IL, interleukin; *IFN*, interferon; CB2R, cannabidiol 2 receptor; DM, dermatomyositis; *TNF*, Tumour necrosis factor; PPARγ, peroxisome proliferator-activated receptor γ. Created in BioRender. Pareek, A. (2026) https://BioRender.com/tp3jvtu.

According to a recent article, lenabasum reduced the amounts of Th2 cells, *IL-31*, *IFN-β*, *IFN-γ*, and aberrant CB2R expression in skin having dermatomyositis. The study showed that *IFN-γ* and *IFN-β* mRNA levels were reduced in DM lesional skin but not in the case of PBMCs. The findings showed that DM lesional skin had greater levels of CB2R expression than both DM PBMCs and healthy control skin (*p* < 0.05). B cells, T cells, dendritic cells, and macrophages all had elevated expression of CB2R in DM skin, with dendritic cells expressing most of it in both DM skin and PBMCs (*p* < 0.05). Studies suggest that CB2R+ immune cells in dermatomyositis skin may modulate cytokine release, including *IL-31*, *IL-4*, *IFN-γ*, and *IFN-β*, as demonstrated in Figure 6 [88]. While these results are intriguing, they are preliminary, and further controlled investigations are required, including additional clinical studies.

### 5.7. Pruritus

CBRs have been recognised as regulators of the neuronal response to itching, suggesting their potential as a target for alleviating itchiness in different skin conditions. Several experimental and early clinical studies have explored the potential of the ECBs in itch regulation. While the results are promising, the evidence base is still limited and heterogeneous. WIN 55,212-2, a CB1R/CB2R agonist, reduced persistent itching in mice by inhibiting *IL-13* and *IL-31* (Figure 4) [61,80]. The relationship between ECBs and itching is complex, with ECB agonists perhaps inhibiting mast cell degranulation and histamine production [61,89,90,91,92,93,94]. According to skin prick studies, falcarinol, an irritant found in ginseng, carrots, and parsley, inhibited CB1R on keratinocytes, causing histamine-induced swelling. As a result of this suppression, proinflammatory cytokines, including *MCP-1* and *IL-8,* increased [93]. Cutaneous sensory nerve fibres express CB1 and CB2 receptors, and pharmacological modulation of these receptors can reduce neuronal excitability and suppress pruritic signalling, thereby contributing to itch relief [94]. Stimulation of CB1R in the CNS has been shown to decrease itching in the central nervous system [95,96].

Schlosburg and colleagues [97] noted that inhibiting neuronal *FAAH* decreased scratching behaviour by preventing the breakdown of AEA and stimulating CB1R activity.

Due to various mechanisms ascribed to CBD, including *FAAH* inhibition, a CB2 inverse agonism (which acts as an antagonist to CB2 agonists), and a TRPV1 agonism, CBD might influence the itch response. However, scientific evidence supporting this specific application is currently limited [98].

### 5.8. Skin Cancer

CBD affects the production of basal and squamous cell carcinoma due to its role in modulating keratinocyte activity [43]. When exposed to continuous ultraviolet B (UVB) radiation, CB1R/CB2R^+^/^+^ mice showed significantly higher rates of tumorigenesis compared to CB1R/CB2R^−^/^−^mice, indicating a receptor-dependent role in skin when exposed to UV, causing cancer development [99]. Gegotek et al. [100] argued in favour of the anticancer effects of CBD. Exposure to UVA and UVB radiation resulted in a marked decrease in ECB receptors, AEA, and 2-arachidonoylglycerol (2-AG) levels in keratinocytes and fibroblasts. The results further suggested that AEA can inhibit *NFkB* activity without relying on CB1/CB2R, indicating the potential pro-apoptotic effects of CBD through undefined mechanisms.

**Figure 7:**
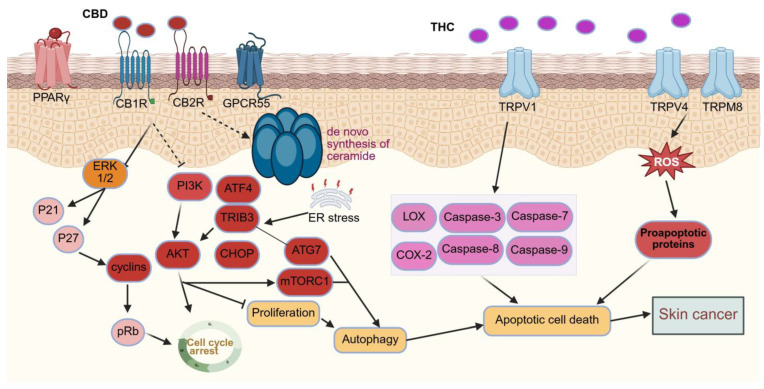
Cannabinoid receptors influence molecular pathways in cancer cells: Cannabinoids like THC and CBD interact with receptors such as CB1R, CB2R, TRPV1, TRPV2, and TRPM8, initiating various intracellular signalling cascades that influence cell fate decisions in cancer cells. By blocking the phosphorylation of retinoblastoma protein (pRb), THC and CBD cause cell cycle arrest by activating CB1 and CB2 receptors, which in turn impact signalling pathways like the *ERK1/2* pathway, which upregulates cyclin-dependent kinase inhibitors (p21 and p27). Through *mTORC1*, the *PI3K*/*AKT* pathway controls autophagy and cell proliferation. Endoplasmic reticulum (ER) stress caused by prolonged activation triggers apoptosis by activating *ATF4*, *TRIB3*, and *CHOP*. To produce *ROS*, CBG interacts with TRPM8, TRPV1, and TRPV2. *ROS* accumulation activates proapoptotic proteins, promoting apoptosis. CBD influences the *LOX* pathway, producing anti-inflammatory and pro-resolving mediators like lipoxin A4, which help resolve inflammation. AEA induces ER-associated apoptosis by upregulating *CHOP10*, activating caspase-12 and caspase-3 in keratinocytes overexpressing *COX-2*. *COX-2* triggers ER stress, leading to apoptotic cell death. By activating caspase-3 and caspase-6 and modulating *MAPKs*, CBD prevents the growth of cancer cells and promotes apoptosis. Arrows indicate activation & regulation, the dotted lines represent non-significant correlations, and the T-shaped symbols denote inhibition. THC, tetrahydrocannabinol; CBD, cannabidiol; CB2R, cannabidiol 2 receptor; *ERK*, extracellular signal-regulated kinase; TRPV, transient receptor potential vanilloid; TRPM, Transient receptor potential cation channel subfamily M member; *ROS*, reactive oxygen species; *LOX*, lipoxygenase; AEA, Anandamide; *CHOP*, C/EBP homologous protein; *MAPK*, mitogen-activated protein kinase; *PI3K*, phosphoinositide 3-kinases; *mTOR*, mammalian target of rapamycin; *COX*, cyclooxygenase. Created in BioRender. Pareek, A. (2026) https://BioRender.com/0f03g6v.

Other studies have demonstrated that activation of CB1- and CB2-dependent pathways can exert antitumour effects in skin cancer models [8,101,102]. In a murine model using PDV.C57 epidermal tumour cells, treatment with WIN-55,212-2, a mixed CB1/CB2 receptor agonist, and JWH-133, a selective CB2 agonist, significantly reduced tumour growth in vivo. This antitumour effect was attributed to the induction of tumour cell apoptosis and inhibition of angiogenesis, as evidenced by decreased expression of vascular endothelial growth factor (VEGF), placental growth factor, and angiopoietin-2. Collectively, these changes led to impaired tumour vascularisation and suppression of tumour progression (Figure 7) [101].

Studies report contradictory findings, with cannabinoid signalling demonstrating both tumour-promoting and tumour-inhibiting effects depending on concentration and biological context [13]. This highlights the complexity of endocannabinoid system regulation in skin oncogenesis. At nanomolar concentrations, endogenous ECBs, particularly under conditions of UVB exposure or chemical carcinogen challenge, may promote tumorigenic signalling in non-melanoma skin cancer. In contrast, exogenously administered cannabidiol at micromolar concentrations has been shown to suppress tumour growth through antiproliferative and pro-apoptotic mechanisms. Similar concentration-dependent effects of cannabinoid signalling have also been reported in melanoma models [13].

Human melanomas contain CB1 and CB2 receptors, and stimulation of these receptors may enhance apoptosis and decrease tumour development in mice. THC reduced cell viability, activated autophagy, and promoted apoptosis in the melanoma cell lines SK-MEL-28, A375, and CHL-1, according to in vitro and in vivo investigations. Compared to temozolomide, THC and CBD were far more effective in decreasing melanoma cell survival, proliferation, and tumour formation in mice with BRAF wild-type melanoma xenografts [102].

However, in melanoma, activating CB1R could encourage tumour development. The absence of CB1R in melanoma cells resulted in reduced expression of p-*Akt* and p-*ERK*, decreased capacity for colony formation and cell migration, and a higher probability of cell cycle arrest in the G1/S phase compared to the control group [101]. It seems that CBD has a variety of effects on the onset of skin cancer. While many studies confirm the anti-neoplastic attributes of CBD, the evidence remains inconclusive [13,43,101,102,103]. Given CBD’s immunomodulatory properties, further research into the impact of CBD on immunosurveillance is crucial for understanding its primary therapeutic effects [12].

## 6. Clinical Trials of CBs

Preclinical data have prompted several clinical studies investigating the potential benefits of cannabinoids for dermatological conditions. Although many of these studies are ongoing or have yet to yield definitive outcomes, topical cannabinoid-based formulations have attracted attention due to their favourable safety profile. Localised cutaneous delivery allows therapeutic effects at the skin level while minimising systemic absorption and psychoactive side effects [104].

### 6.1. Topical CBs Mechanistic Action

Proinflammatory cytokines, which include *IL-6* and *IL-17,* are reduced by the topical administration of Δ9-THC and CBD. Interestingly, CBD pre-treatment has led to an increase in *IL-10*, which is recognised for having anti-inflammatory characteristics [105]. These impacts on the immune system seem to occur regardless of CBs’ signalling pathways [106]. Several investigations [107,108,109,110,111,112,113,114,115,116,117,118,119,120,121,122,123,124,125] have focused on topical CBs’ formulations, and their findings are summarised in Table 1.

#### 6.1.1. Psoriasis

Several small-scale studies have evaluated the topical application of cannabinoids in psoriasis, with generally positive but preliminary outcomes. In a retrospective cohort, CBD ointment applied twice daily for three months improved PASI scores and skin hydration without reported adverse events [107]. A case report also documented rapid resolution of lesions following use of THC-containing topical preparations [108]. In a randomised single-blind trial, two formulations containing CBD and THC were found to reduce disease severity and improve the quality of life, with no alterations in blood chemistry [109]. More recently, in a double-blind study, CBD oil was well tolerated but did not significantly reduce the severity of psoriasis. Temporary improvements in sleep onset and itch alleviation suggest that more studies using larger dosages and longer durations are necessary [110].

As noted above in Section 5.1, CBs appear beneficial in psoriasis. Taken together, the clinical studies indicate potential anti-inflammatory and antiproliferative effects of cannabinoids in psoriasis.

#### 6.1.2. Atopic Dermatitis

Multiple clinical investigations have examined the role of topical cannabinoids and related compounds in AD, with encouraging but heterogeneous findings. In a small pilot study, topical adelmidrol (an anti-inflammatory ethanolamide derivative of azelaic acid) achieved complete or near-complete remission in most participants within four weeks [111]. A large multinational observational trial involving over 2400 patients reported that PEA-based emollients reduced visible symptoms such as erythema and scaling, while also improving sleep quality and reducing reliance on corticosteroids [112]. Additional controlled studies of PEA/AEA-containing moisturisers demonstrated improvements in dryness, itching, and transepidermal water loss, although statistical significance was not consistent across all endpoints [113].

Several CBD-based formulations have also been tested. A recent randomised trial of JW-100, a CBD-aspartame cream, demonstrated significant ISGA score reductions after two weeks, while other CBD products, such as shampoos and creams, showed modest improvements in inflammation and pruritus, though some lacked placebo controls [114,115,116]. Non-steroidal creams containing PEA and newer supplements such as Levagen+ have also been reported to reduce eczema severity and pruritus in randomised and open-label settings [117,118]. AD symptoms have also been reported to be reduced by topical use of a 1% CBD gel or a combination of ginger extract and CBD [119,120].

Collectively, these studies suggest that cannabinoids—particularly CBD and PEA—may provide symptomatic relief in AD through anti-inflammatory and barrier-restoring effects. However, differences in formulations, trial design, and endpoints limit the comparability of findings, and most studies remain small or uncontrolled. Robust, placebo-controlled trials are still required to establish the efficacy, safety, and long-term tolerability of cannabinoid-based therapies in AD.

#### 6.1.3. Pruritus

Several clinical investigations have explored the antipruritic effects of topical endocannabinoid-related compounds. In a preliminary open-label study, an emollient containing AEA and PEA (palmitoylethanolamide) reduced xerosis in over 80% of patients with uremic pruritus and provided complete itch relief in nearly 40% of cases [88]. Subsequent observational studies using PEA-based creams in patients with prurigo, lichen simplex, and chronic pruritus similarly reported significant reductions in itch intensity and high patient satisfaction [121]. Larger prospective trials, including a study of 100 individuals with chronic pruritus, demonstrated improvements in pruritus severity and quality-of-life measures, although some comparisons with control formulations revealed only modest benefits [122].

As mentioned above, preclinical evidence shows that CBD and PEA can improve skin barrier function and reduce inflammation in AD. Subsequent clinical trials have likewise explored CBD-based treatments, noting improvements in itch and lesion severity. The safety profile has generally been favourable, with only mild and transient local reactions occasionally reported. However, the available studies are limited by their open-label or uncontrolled design, small sample sizes, and short follow-up. Rigorous randomised controlled trials are required to confirm efficacy, determine optimal dosing, and evaluate long-term outcomes in diverse pruritic conditions.

#### 6.1.4. Acne Vulgaris

Topical cannabinoids have also been investigated in acne, primarily for their anti-inflammatory and sebostatic effects. In a small single-blind trial, application of a cream containing cannabis seed extract twice daily significantly reduced erythema and sebum production without causing irritation or allergic reactions [45]. More recently, a large phase II clinical trial evaluated BTX 1503, a cannabidiol-based topical solution, in over 300 patients with moderate to severe acne. Treatment was associated with a dose-dependent reduction in inflammatory lesion counts and overall acne severity compared with placebo, and the formulation was well tolerated [123].

Assessing the safety and efficacy of microneedling with CBD and hempseed oil as a therapy for moderate to severe acne in people aged 22 years or older is the main objective of this study. Because microneedling does not harm sebaceous glands or alter the skin microbiota, it may be a well-tolerated therapy option for acne sufferers [124].

Based on the above data, preliminary trials of topical CBD have shown improved acne lesions. However, despite promising early data, evidence is limited to a small number of trials, and long-term efficacy, safety, and comparative effectiveness against standard treatments such as retinoids or antibiotics remain to be established.

#### 6.1.5. Epidermolysis Bullosa

Epidermolysis bullosa is a rare blistering skin condition that is difficult to manage due to skin fragility and repetitive wound healing, resulting in itching, discomfort, limited mobility, and recurring infections [125]. Two studies [112,125] examined topical CBD in six paediatric patients (ages 6 months to 10 years). All patients reported better wound healing, less need for narcotic analgesics, and a minimum 50% decrease in blisters.

### 6.2. Oral and Other Route CBs Mechanistic Action

Recent research has elucidated various mechanisms by which oral CBs exert their influence. One such mechanism involves systemic sclerosis (SSc) fibroblasts, which exhibit elevated levels of CB2R. Oral CB2R agonists target these receptors to decrease the production of *TGFb* and collagen, thereby restraining the fibrosis commonly associated with SSc [126]. The ECS facilitates innate immune response resolution by CB2R agonist stimulation, which develops pro-resolving lipid mediators such as lipoxin-A4, lipoxin-B4, resolvin-D1, and resolvin-D3. It has been shown that activating CB2Rs in lymphoid tissue inhibits immune cells’ cytokine release, reducing inflammation [126]. The potent anti-inflammatory effect of CB2R agonists stems partly from their ability to block leukotriene B4, a chemical that attracts neutrophils, thereby facilitating the efficient removal of inflammatory triggers by inhibiting pro-inflammatory prostaglandins like prostaglandin E2, thromboxane B2, and prostaglandin F2α, which hinder phagocytosis [127]. Many investigation studies have been done on oral and other administrations of CBs, which are summarised in Table 2.

According to recent studies, patients with dermatomyositis (DM) may benefit from oral CB2R agonists since they regulate their immune systems. In a recent study, proinflammatory cytokines, including *TNF-α*, *IFN-β*, and *IFN-α*, have been found to be reduced in individuals treated with ajulemic acid (lenabasum), a CB2R agonist [128].

**Table 2:** Summarised data of oral and other route CB investigation studies.

Condition	Cannabinoids	Parameters of Assessment	Research Design and No. of Patients (*n*)	CBD Therapy Schedule	Outcomes	Adverse Effects	References
**Dermatomyositis**							
	Lenabasum	(a) CDASI score (b) Skin biopsy	OLE study, double-blind, single-centre (*n* = 22)	Oral lenabasum for 3 years	By week 68, patients exhibited reductions in CDASI and Severity Index activity score (−21.8) Patient Skin Activity VAS (−3.0), and Skindex-29 (−28.0) from OLE baseline	Mild dizziness, fatigue, dry mouth, nasopharyngitis, DM worsening	[129,130]
	Lenabasum	(a) TSI (b) CDASI	multicentre, randomised, double-blind, placebo-controlled, phase III study completed (*n* = 150)	Oral lenabasum (20 mg b.i.d.) for 52 weeks	By week 52, the CDASI score decreased by 15 points. 82.3% of patients achieved ≥20% improvement. Overall disease activity reduced by −2.6 ± 1.90 points.	Mild AEs: dizziness and diarrhoea in 5 patients (25%), DM flare in 2 patients (10%)	[131,132]
**Systemic Sclerosis**							
	Lenabasum	(a) ACR-CRISS (b) MRSS (c) PGA (d) PtGA (e) PROMIS-29 (f) 5-D itch scale (g) Skin biopsy	Randomised, double-blind, placebo-controlled (*n* = 42)	Oral lenabasum: 5 mg or 20 mg q.d., or 20 mg b.i.d. for 4 weeks, followed by 20 mg b.i.d. for 8 weeks	Median ACR-CRISS score at week 16: 0.33 (vs. 0.00 in placebo). Reduced expression of inflammatory genes.	AEs: 60% (placebo) vs. 63% (lenabasum). Most common: nausea, vertigo. No sign of toxicity.	[126]
	Lenabasum	(a) ACR-CRISS (b) MRSS (c) PGA (d) PtGA	Randomised, double-blind, placebo-controlled (*n* = 42)	Oral lenabasum: 5 mg or 20 mg q.d., or 20 mg b.i.d. for 4 weeks, followed by 20 mg b.i.d. for 8 weeks	Significant improvement in ACR-CRISS scores vs. placebo at week 16 (*p* = 0.044).	Mild fatigue (14%), mild/moderate URI (11%), dizziness (6%)	[133]
	Lenabasum	(a) ACR-CRISS (b) MRSS (c) PGA (d) PtGA (e) Itch questionnaire	Open-label, double-blind, placebo-controlled (*n* = 365)	Oral lenabasum (20 mg b.i.d. for 52 weeks)	CRISS score of drug vs. placebo 0.888 vs. 0.887 (*p* = 0.4972) High CRISS scores Improvement in MRSS Lower decline in forced vital capacity	None reported	[134]
	Cannabis	(a) Photographic	Case report (*n* = 1)	Inhaled cannabis (30 g/day)	Symptom improvement Resolution of Raynaud’s phenomenon and dyspnoea	None reported	[135]
**Pruritus**							
	HU210 (Cannabinoid receptor)	(a) VAS (b) Blood flow (c) Magnitude estimation	Two studies: 1. Iontophoresis (double-blind) *n* = 12 2. Microdialysis (single-blind) *n* = 6	HU210 (CB agonist) applied via skin patch (study 1) and intradermally (study 2)	relief from itching. Reduced neurogenic flare reactions. Reduced skin blood flow (*p* < 0.003 and *p* < 0.03).	Not reported	[93]
**Psoriatic Arthritis**							
	CBD	VAS	Placebo-controlled, randomised *n* = 129	10 mg CBD tablets once daily for 2 wks to b.i.d. for 3 & 4 wks	Less relief from pain intensity	Not reported	[136]
**Epidermolysis Bullosa**							
	Epidyolex (CBD)	EBDASI VAS	Interventional study *n* = 10	Orally taken 3 times a day;	NA	NA	[137]
	Δ9-THC CBD	VAS	Observational study *n* = 3	Sublingually for 3 months	Relief from pain Pruritus decreased	Appetite increased; Sense of time altered	[138]

Abbreviations: CDASI, Cutaneous Dermatomyositis Disease Area and severity index; TSI, Total skin irritation; ACR-CRISS, American College of Rheumatology Combined Response Index in diffuse cutaneous Systemic Sclerosis; MRSS, Modified Rodman skin score; PGA, Physician global assessments; PtGA, Patient global assessments; PROMIS-29, Patient-reported outcomes measurement information system 29-item; 5-D itch, Five-Dimensional Itch Scale; VAS, Visual Analogue Scale; URI, Upper Respiratory Infection; AE, Adverse Event.

#### 6.2.1. Dermatomyositis

Oral administration of lenabasum has been tested in dermatomyositis with encouraging results. Building on these observations, the open-label extension (OLE) of the NCT02466243 trial demonstrated that lenabasum reduced CDASI activity scores by 21.8 points at week 68, with 58.3% of patients maintaining stable disease for at least three years after discontinuing treatment, significantly higher than the 20% in controls. While that study established CB2R activation as a key anti-inflammatory mechanism, it did not account for heterogeneity in patient response. Our analysis addresses this gap, revealing that responders exhibited higher baseline CB2R expression, suggesting that receptor availability may drive treatment efficacy and long-term disease stability [129,130]. Extension and phase III studies have since reported improvements in skin lesions, pruritus, and overall disease activity [131,132].

Overall, oral CB2R agonists such as lenabasum appear to modulate immune pathways relevant to dermatomyositis and provide symptomatic relief. However, despite promising phase II outcomes, phase III data are still emerging. The therapeutic role of cannabinoids in dermatomyositis, therefore, remains preliminary and requires confirmation in larger, well-controlled trials.

#### 6.2.2. Sclerosis

Growth factors such as platelet-derived growth factor, connective tissue growth factor, and *TGF-β* are blocked by this synthetic cannabis. In phase 2 studies, patients with diffuse cutaneous systemic sclerosis showed improved symptom index scores when treated with oral lenabasum [133]. Currently, a Phase 3 clinical investigation is underway [134].

A recent case report also described remission of Raynaud’s phenomenon and dyspnoea following inhaled cannabis use, though such anecdotal evidence cannot establish causality [135]. Apart from a closely monitored safety trial where dizziness was the most common side effect, lenabasum treatment was linked to improvements in patients’ overall condition, skin symptoms, and daily activities. Ajulemic acid is a synthetic THC derivative that binds to the CB2 receptor. It is also referred to as lenabasum or JBT-101. Furthermore, treatment attenuated the development of bleomycin-induced skin fibrosis in animal models [128].

These findings indicate that CB2R-targeted therapies may hold promise in modulating fibrosis and systemic inflammation in systemic sclerosis. Nevertheless, the variability of clinical endpoints, modest effect sizes, and reliance on small or open-label cohorts highlight the urgent need for conclusive phase III trial data to determine whether cannabinoids can be integrated into standard care for this condition.

#### 6.2.3. Pruritus

The antipruritic effects of oral and peripherally applied cannabinoid agonists have also been evaluated. In human models of histamine-induced itch, administration of HU210, a potent CB1R/CB2R agonist, reduced flare reactions, skin blood flow, and subjective itch intensity [93]. These effects were independent of antihistamine pathways, suggesting a distinct mechanism of action.

Although these findings support the potential of cannabinoid receptor agonists for refractory pruritus, the data remain limited to experimental models and small preliminary studies.

#### 6.2.4. Psoriatic Arthritis

In contrast to other illnesses, the impact of cannabinoids on psoriatic skin symptoms has not been thoroughly studied in the literature [136]. Only CBD was investigated; however, it had inconsistent outcomes when compared to a placebo and was unable to reduce the discomfort associated with psoriatic arthritis.

Vela et al. [136] examined how 136 people with psoriatic arthritis or hand osteoarthritis experienced pain intensity after receiving oral CBD medication for 12 weeks (20–30 mg/day). CBD did not affect sadness and anxiety ratings, sleep quality, or pain reduction when compared to a placebo. When compared to a placebo, CBD was well-tolerated and did not statistically significantly increase side events.

#### 6.2.5. Epidermolysis Bullosa

Three patients with epidermolysis bullosa, aged 36–61, were investigated for the potential therapeutic benefits of phytocannabinoids (CBD and Δ9-THC) [137]. All individuals received sublingual administration of Δ9-THC (13 mg/mL) and CBD (20 mg/mL). An improvement in pain and pruritus was the primary outcome that was examined, and it was observed after just one month of therapy. One patient was able to stop using topical morphine and amitriptyline after two years of therapy with the sublingual CB regimen by adding topically applied Δ9-THC−CBD oil (1 mg CBD with 0.65 mg Δ9-THC). The patient also stayed on the sublingual CB regimen. Despite taking numerous opioids, a patient with generalised severe recessive dystrophic epidermolysis bullosa continued to have excruciating pain; nevertheless, after one week of sublingual CBD−Δ9-THC combo oil, the patient reported a 40% decrease in pain [138].

## 7. Challenges for Cannabinoid Applications

Despite the abundance of commercially available topical products and oral CB preparations approved by the United States Food and Drug Administration (FDA), their widespread medical use has been hampered by an insufficient understanding of their processes and the physicochemical properties of CB compounds [104]. Challenges related to solubility, stability, permeation, and metabolism contribute to significant variability in pharmacokinetics among individuals. Moreover, the lipophilic nature of CBs leads to irregular bioavailability when administered orally or transmucosally [139]. These substances are prone to degradation, especially when exposed to fluctuations in light and temperature, necessitating the use of suitable oil bases to minimise oxidation and contaminants. Swift absorption and metabolism following systemic or topical administration might hinder their desired effects [140].

Limited research has been conducted on how the carrier influences the penetration characteristics of topical CB formulations, both in laboratory settings and in living organisms. Challenges such as low solubility in water and quick breakdown in living organisms have posed significant hurdles for topical application. Furthermore, the presence of various inherent crystalline forms in numerous CBs compounds adds another layer of complexity to the formulation. These polymorphisms can cause alterations in molecular structures, impacting both solubility and degradation [139,141]. Effective drug delivery methods that can preserve the required drug concentration in solution form while retaining the formulation’s stability are essential for the practical application of CBs in dermatology [104].

## 8. Translational Challenges and Emerging Delivery Approaches

### 8.1. Pharmaceutical and Biopharmaceutical Design Considerations

The physicochemical and pharmacokinetic constraints discussed in the preceding section present substantial barriers to the clinical translation of cannabinoids in dermatology. Achieving consistent therapeutic concentrations in cutaneous tissue remains challenging due to poor aqueous solubility, high lipophilicity, chemical instability, and interindividual variability in absorption and metabolism [34,139,140].

Topical administration requires effective penetration across the stratum corneum while maintaining localised dermal retention. Although the lipophilic character of cannabinoids facilitates interaction with the epidermal lipid matrix, excessive hydrophobicity may restrict diffusion into deeper viable skin layers [3,48]. Therefore, formulation development must balance cutaneous retention with controlled permeation to maximise local activity while minimising unintended systemic exposure.

In addition, variability inherent to plant-derived preparations complicates standardisation. Differences in cannabinoid purity, extract composition, and minor constituent profiles may influence pharmacodynamic outcomes and reproducibility [23,141].

To address these limitations, pharmaceutical-grade standardised cannabis extracts and raw materials have been developed. These include chemovar-defined cannabis sources [142] and GMP-manufactured full-spectrum extracts that ensure consistent CBD and terpene profiles across production batches [143]. In addition, regulated cannabinoid medicines such as Epidiolex (purified cannabidiol) and Sativex (a standardised THC/CBD extract) exemplify how controlled manufacturing and standardisation can enable reproducible cannabinoid formulations suitable for clinical use [144].

From a translational perspective, defined drug loading, encapsulation efficiency, stability profiling, and reproducible manufacturing are critical requirements for advancing cannabinoid-based products toward regulated clinical development [33,48].

### 8.2. Nanotechnology-Enabled Cannabinoid Delivery Systems

Nanotechnology-based platforms have been increasingly investigated to overcome solubility, stability, and permeability limitations associated with cannabidiol and related cannabinoids. Nano-scale carriers such as liposomes, nanoemulsions, polymeric nanoparticles, nanostructured lipid carriers, hydrogels, and hybrid systems have been explored to improve controlled release and targeted skin deposition [33,106]. These platforms may increase local drug concentration at pathological sites while reducing systemic distribution [145].

Lipid-based nanocarriers, including solid lipid nanoparticles and nanostructured lipid carriers, have demonstrated improved dermal penetration, enhanced entrapment efficiency, and protection against oxidative degradation in experimental systems [146]. In models of non-melanoma skin cancer, multifunctional nano-lipid carrier gels incorporating CBD have shown enhanced localised delivery compared with conventional formulations, supporting the relevance of carrier architecture in modulating tissue exposure [146].

Nanoemulsion-based systems have also been developed to enhance CBD solubilisation and cutaneous distribution. Encapsulation of CBD within oil-in-water nanoemulsions and nanoemulsion-filled hydrogels improved structural stability and dermal absorption characteristics compared with non-nano formulations [147]. Similarly, micelles-in-liposome systems prepared via proliposomal approaches have demonstrated favourable structural properties and enhanced skin penetration profiles for CBD delivery [148]. Microemulgels optimised for local dermatological application have further shown controlled-release behaviour and suitable rheological characteristics for cutaneous administration [149].

More recent full-thickness human skin investigations further demonstrate that formulation-dependent differences significantly influence CBD partitioning across the stratum corneum, viable epidermis, and dermis. Quantitative biodistribution analysis highlights that optimised colloidal systems can enhance dermal deposition while limiting systemic permeation, highlighting the importance of rational carrier design in dermatologic applications [3]. Such findings suggest that engineered colloidal systems may improve dermal targeting efficiency without necessarily increasing systemic exposure.

Emerging nanoemulsion systems have also demonstrated functional biological relevance in human skin models. In a 2025 ex vivo human skin study, CBD-loaded nanoemulsions enhanced dermal penetration and reduced inflammatory biomarkers, including IL-6 and MMP-1, while restoring procollagen type I levels under stress conditions [150]. Although preclinical in nature, these data support the concept that nano-enabled delivery can influence both pharmacokinetic and biological outcomes within viable skin layers.

Additional advanced delivery systems—including β-cyclodextrin cryogels [151], nanocomposite cryogel carriers [152], Pickering emulsions [153], alginate-based hydrogels for wound healing [154], and organosilane-assisted transdermal systems [155]—have demonstrated sustained release behaviour, enhanced stability, and improved penetration profiles in experimental settings.

Collectively, these studies indicate that nano-enabled platforms can meaningfully modify CBD stability, dermal deposition, and release kinetics. The principal nanoformulation strategies investigated for dermatological cannabinoid delivery are summarised in Table 3.

However, it is important to emphasise that the majority of available data derive from in vitro, ex vivo, or preclinical models. Robust randomised clinical trials evaluating nano-CBD formulations in specific dermatologic diseases remain limited.

### 8.3. Emerging Transdermal and Hybrid Platforms

Beyond conventional nanoemulsions and lipid carriers, hybrid and polymeric systems are being explored to further optimise cutaneous and transdermal delivery.

Hydrogel-based systems incorporating CBD have demonstrated sustained release and favourable biocompatibility in wound-healing models [154]. Electrospun films and nanoparticle-integrated patches have shown enhanced dermal penetration and controlled-release behaviour in experimental studies [155,156].

Transdermal enhancement strategies may offer advantages for lipophilic molecules by improving percutaneous transport and bypassing first-pass metabolism [157]. However, enhanced penetration must be balanced against potential systemic exposure, particularly for cannabinoids with central nervous system activity.

Future translational progress will require rigorous pharmacokinetic characterisation in human skin, standardised formulation protocols, and comparative clinical evaluation against established dermatologic therapies. Bridging the gap between experimental nanocarrier development and clinical dermatology remains a critical priority.

Despite these advances, concerns remain. Enhanced skin penetration may also increase systemic absorption, raising the risk of unwanted psychoactive effects. Furthermore, most current evidence derives from preclinical or pilot studies, with limited data from large-scale clinical trials in dermatology. Thus, while nanoformulations hold clear promise for improving cannabinoid therapy, rigorous evaluation of safety, long-term tolerability, and comparative effectiveness against existing dermatological treatments is still required [104]. The key nanoformulation strategies investigated for cannabinoid delivery in dermatology are summarised in Table 3.

**Table 3:** Summary of cannabinoid-based nano formulation strategies investigated for dermatological applications.

Nano Formulations	Composition	Method	Key Findings	Dermatological Application/Intended Use	References
**Lipid based Nanocarriers**					
Nanostructured lipid carrier gel	CBD, Gelucire 53/15, coconut oil, Tween 80, Transcutol	Multiple emulsification	Less irritating Improved skin retention Well tolerated	Skin cancer	[146]
Liposomes	CBD, soy phosphatidylcholine, tween 80, trehalose, sucrose or lactose.	Spray-drying/slurry	Improved skin permeation Sustained release Increased epidermal absorption	Cutaneous CBD delivery	[148]
Solid lipid nanoparticles	Ethyl cellulose, triethyl citrate, lipids (cetyl alcohol, lauric acid), CBD	Rotary evaporation and centrifugation	↓ Psoriasis severity ↓ IL-17A, PASI, IL-6, IL-8 EE (%) = 85%	Psoriasis; enhanced skin penetration	[158,159]
**Emulsions and Colloidal Systems**					
Nanoemulsions and hydrogels	CBD; chitosan (hydrogel)	High-pressure homogenization; Phase inversion	Increased stability, penetration, and CBD release	Wound healing; anti-inflammatory	[147]
Microemulsion/Microemulgel	CBD, isopropyl myristate, Solutol HS15, Transcutol P	Aqueous titration	Controlled dermal absorption Suitable viscosity	Local skin delivery	[149]
Nanoemulsion	CBD, Brij^®^ O10 (polyoxyethylene (10) oleyl ether), olive oil	Phase inversion temperature method.	Reduced inflammation Improved extracellular matrix degradation	Particulate matter exposed skin	[150]
Pickering emulsions	CBD, chitosan, gum Arabic, olive oil	High-speed homogenisation	High stability (no phase separation) High CBD encapsulation Controlled skin delivery	Enhanced retention in the stratum corneum.	[153]
Colloidal VESIsorb^®^ system	CBD, Propylene glycol	Proprietary colloidal tech	Superior skin permeation compared to conventional formulations.	Enhanced epidermal/ dermal delivery	[3]
**Polymeric Hydrogels, Cryogels and Scaffolds**					
Polysaccharide cryogels	CBD, 2-hydroxyethyl cellulose, β- cyclodextrin, TEA	Precipitation; Photochemical crosslinking	Bi-phasic release High loading efficiency SD = 36–46 ± 4 Particle size 212–350 nm	Skin cancer; cutaneous T-cell lymphoma	[151]
Nanocomposite cryogel (HEC/PM)	PEO101-b-PPO56-b-PEO101, HEC, Polyethyleneglycol diacrylate, N,N’-methylenebisacrylamide, CBD	Cryogenic treatment; Photochemical crosslinking	Good mechanical properties High GF yield 80–85% Sustained release	Topical delivery; Cutaneous lesions and Antineoplastic activity	[152]
Hydrogel	Sodium Alginate, CBD, Zinc sulfate	Ionic crosslinking	Biocompatible Controlled inflammatory infiltration Promote collagen deposition and granulation tissue.	Wound healing and Skin tissue engineering	[154]
Microspheres	CBD, PLGA, PVA, Tween 20, Gelatin	Single emulsion (O/W) solvent evaporation	Reliable scaffold Osteogenesis support	Bone regeneration; skin tissue engineering	[160]
**Films and Transdermal Patches**					
Organosilane polyvinyl alcohol films	CBD, polyvinyl alcohol mats	Electrospinning	Stable for 14 weeks Enhanced penetration	Transdermal drug delivery	[155]
Crosslinked chitosan/ZnO NPs based patches	CBD, Fungal chitosan, Potassium hydroxide, Zinc acetate dihydrate	Precipitation	Sustained release High skin biocompatibility	Transdermal therapy (unspecified dermatoses)	[156]
3D-printed sodium alginate films	Sodium alginate, CBG, CBD, Pluronic-F127	Bioprinting	Good shape fidelity and water retention. High DL and EE (97–99%)	Topical delivery; wound healing	[6]
**Metallic and Inorganic Nanoparticles**					
Metal oxide nanoparticles	CBD, Zinc nitrate, polyvinylpyrrolidone, ferric nitrate nonahydrate, sodium hydroxide	Precipitation	No cytotoxicity in keratinocytes	Transdermal drug delivery; cancer	[161]
Gold and silver nanoparticles	Silver nitrate, polyvinylpyrrolidone, chloroauric acid trihydrate, CBD	Green synthesis (Microwave hydrothermal)	Ag NPs (4.8 nm), Au NPs (8.4 nm) Low cytotoxicity Au NPs enhanced anticancer potential	Transdermal drug delivery; Cancer therapy	[162]

Abbreviations: CBD, Cannabidiol; IL, Interleukin; TEA, Triethanolamine; EE, Entrapment efficiency; PASI, Psoriasis Area and Severity Index; PVA, Polyvinyl alcohol; PLGA, Polylactic-co-glycolic acid, O/W, Oil in water; SD, Swelling degree; HEC, Hydroxyethyl cellulose; PM, Polymeric micelles; Gold (III) chloride trihydrate; ZnO, Zinc oxide; DL, Drug loading; CBG, Cannabigerol, Ag NPs, Silver Nanoparticles.

### 8.4. Clinical Translation and Future Directions

Although nanotechnology and advanced delivery platforms provide rational solutions to the physicochemical limitations of cannabinoids, clinical translation in dermatology remains limited by insufficient integration of formulation science with controlled human evaluation. Most available clinical studies are small, exploratory, and lack standardised characterisation of the applied formulation, making interpretation of therapeutic outcomes difficult.

Human pharmacokinetic evidence further highlights the need to clearly distinguish systemic transdermal delivery from localised topical therapy. In an exploratory study in healthy adults, application of a transdermal formulation containing 100 mg CBD and 100 mg tetrahydrocannabinol resulted in relatively low systemic exposure, with a mean CBD Cmax of approximately 0.576 ng/mL and marked interindividual variability [163]. Importantly, this study was not disease-specific, but it demonstrates that transdermal administration yields limited plasma concentrations compared to oral delivery, reinforcing the rationale for targeting local cutaneous effects in dermatologic indications rather than pursuing systemic exposure. Recent human skin biodistribution investigations have similarly emphasised the importance of finite dose application models and quantitative dermal distribution assessment to better predict in vivo behaviour [3]. However, correlations between dermal tissue concentrations, modulation of inflammatory biomarkers, and clinical outcomes remain insufficiently defined.

Future clinical research should therefore prioritise:

- Standardised formulation, characterisation and stability profiling;
- Finite dose application protocols reflecting real-world use;
- Quantitative dermal biodistribution and pharmacokinetic pharmacodynamic integration;
- GMP-compliant, reproducible manufacturing;
- Comparative evaluation against established dermatologic therapies.

Bridging mechanistic dermatologic insight with rigorous pharmaceutical development will be essential for advancing cannabinoid-based formulations toward reproducible, evidence-grounded clinical application in skin disease [3,48].

## 9. Conclusions

This review highlights the current mechanistic, preclinical, and emerging clinical evidence supporting the potential role of cannabinoids in dermatological disorders. By interacting with CB1 and CB2 receptors, TRP channels, and PPARγ, these regulate keratinocyte proliferation, sebocyte function, fibroblast activity, immune modulation, and skin barrier repair, suggesting applications across inflammatory, autoimmune, and neoplastic skin disorders.

Encouraging preclinical and early clinical data indicate benefits in psoriasis, atopic dermatitis, acne, pruritus, systemic sclerosis, and skin cancers. However, most studies remain limited to small cohorts, uncontrolled designs, or in vitro and animal models, restricting translation into clinical practice. Contradictory results, particularly in oncology, where cannabinoids may demonstrate both tumour-promoting and tumour-inhibiting effects, highlight the complexity of the endocannabinoid system and the need for cautious interpretation.

A central barrier to clinical advancement remains the physicochemical profile of cannabinoids, characterised by poor aqueous solubility, lipophilicity, instability, and variable skin penetration. Nanotechnology-enabled platforms, including lipid-based carriers, nanoemulsions, micellar systems, and advanced hydrogel matrices, offer rational strategies to enhance stability, dermal deposition, and controlled release. However, improved delivery must be accompanied by rigorous assessment of long-term safety, dermal pharmacokinetics, and the balance between local efficacy and unintended systemic exposure.

Future progress requires integration of standardised formulation development, finite-dose biodistribution analysis, pharmacokinetic–pharmacodynamic correlation, and well-designed randomised controlled trials using reproducible manufacturing practices. Comparative evaluation against established dermatologic therapies will be essential to define the therapeutic positioning of cannabinoid-based interventions.

In summary, cannabinoids represent a biologically plausible yet clinically evolving therapeutic class in dermatology. Advancing their role in patient care will depend on coordinated progress in mechanistic understanding, pharmaceutical design, and structured clinical validation.

## Figures and Tables

**Table 1:** Summarised data of topically applied cannabinoids investigation studies.

Condition	Cannabinoid	Parameters of Assessment	Research Design and No. of Patients (*n*)	CBD Therapy Schedule	Outcomes	Adverse Effects	References
**Psoriasis**							
	CBD	(a) Hydration and TEWL (b) Elasticity (c) Photographic (d) PASI	Retrospective cohort (*n* = 20)	CBD ointment applied topically twice daily for 3 months	Significant reduction in psoriasis patches. PASI scores improved by day 90 (*p* < 0.001). Increased hydration and improved TEWL.	None reported	[107]
	THC	(a) Photographic	Case report (*n* = 1)	Hemp-infused soap containing THC (5 mg/mL) and hair oil (5 mg/mL) containing dissolved THC distillate	Complete resolution of lesions within 2 weeks. Maintenance therapy with weekly use thereafter.	None reported	[108]
	CBD + THC	(a) PASI (b) DLQI (c) PDI (d) Blood chemistry	Randomised, single-blind cohort study (*n* = 51)	Topical cream (1.25 mg CBD + 1.35 mg THC) for 56 days	Significant reduction in disease intensity. Improved quality of life scores. No change in blood profile.	None observed.	[109]
	CBD	(a) PASI (b) QOL	Double-blind, randomised (*n* = 28)	CBD oil (60 mg/day)	Reduction in itch by 8 weeks Well-tolerated	Mild to moderate	[110]
**Atopic dermatitis**							
	Adelmidrol	(a) IGA score (b) Pruritus	Pilot study, double-blind (*n* = 20)	Topical adelmidrol b.i.d. for 4 weeks	16 of 20 participants achieved complete resolution of symptoms	None observed.	[111]
	N-PEA	(a) SCORAD (b) Questionnaires	Observational, non-controlled, multinational, multicentre cohort (*n* = 2456)	Topical N-PEA, twice daily for 6 weeks.	Notable reduction in scaling, dryness, erythema, excoriations, lichenification, and pruritus	Burning Erythema Pruritus	[112]
	PEA AEA	(a) Eczema Area and Severity Index (b) Hydration and TEWL	Monocentric, double-blind, randomised, comparative trial (*n* = 60)	Emollient PEA/AEA cream	Improvement in dryness, itching, and scaling. Increased hydration and reduced TEWL.	Not reported	[38]
	CBD	(a) POEM (b) QOLHEQ	Open-label Cohort (*n* = 14)	CBD applied topically twice daily for 2 weeks.	POEM score reduced from 16 ± 1.35 to 8.25 ± 1.80 (*p* < 0.0007). QOLHEQ reduced from 20.9 ± 2.06 to 8.37 ± 1.61 (*p* < 0.004).	Not reported	[113]
	JW-100	(a) ISGA score	Double-blind, placebo-controlled, parallel assignment (*n* = 126)	Topical CBD (JW-100) for 14 days	Significant ISGA score reduction (*p* = 0.042).	Not reported	[114]
	CBD	(a) Videodermatoscope (b) Questionnaires	Observational study (*n* = 50)	CBD shampoo (0.075%) daily for 2 weeks	Reduced vascular inflammation and capillary distortion by day 14	Not reported	[115]
	BTX 1204	(a) IGA	Double-blind, placebo-controlled interventional (*n* = 200)	Topical formulation for 84 days	No significant improvement	Not reported	[116]
	PEA	(a) Documentation (b) Scoring	Single-blinded, split-body, randomised trial (*n* = 43)	PEA-containing nonsteroidal cream for 2–4 wks	73% patients preferred to use PEA-containing cream over the moisturiser	Mild, transient stinging (no serious AEs)	[117]
	Levagen+	(a) SA-EASI (b) DLQI (c) POEM (d) Pruritus Numerical rating Scale	Randomised, double-blind trial, Interventional (*n* = 72)	Topical Levagen+ vs. standard moisturiser for 4 weeks	Significant reduction in SA-EASI. Significant reduction in redness and dryness.	Not reported.	[118]
	CBD	(a) HOME (b) RECAP (c) C/IDQOL	Monocentric, prospective, interventional (*n* = 100)	oil-in-water emulsions (BNO 3732 and BNO 3731) topically for 12 wks	Improvement in pruritus Reduced erythema and dryness	None reported	[119]
	CBD	(a) NRS	Randomised (*n* = 44)	Topically for 5 days oil-in-water emulsion	Improved AD symptoms Itch intensity reduced	None reported; well tolerated	[120]
**Pruritus**							
	AEA N-PEA	(a) Standard VAS (b) Questionnaires	Observational, preliminary (no placebo control) (*n* = 21)	Topical AEA/N-PEA (Physiogel AI^®^), b.i.d. for 3 weeks	Complete reduction in xerosis in 81% patients Pruritus reduced in 38% (*p* < 0.001).	Not reported	[89]
	N-PEA	(a) VAS (b) Clinical examination (c) Photo documentation	Open-label, observational (*n* = 2456)	Topical N-PEA for 6 months	Reduction in itching. No significant change in aquagenic or cholestatic pruritus.	Not reported	[121]
	N-PEA	(a) VAS (b) HRQOL	Randomised, controlled, noninterventional, open-label prospective study (*n* = 35)	Topical N-PEA, b.i.d. for 2 weeks	Following a 2-week therapy, Significant reduction in VAS (*p* < 0.001). Improved HRQOL scores.	Pruritus, stinging, scaling, erythema (mild)	[122]
**Acne**							
	BTX 1503	(a) Lesion counts (b) IGA	Open-label, single-arm study (*n* = 368)	Topical formulation (BTX 1503) b.i.d. for 28 days	Safe and well-tolerated Reduction in acne lesion counts	None reported	[123]
	Hemp oil THC	(a) Facial blemishes Improvement of skin tone	Observational study, Case–control (*n* = 50)	40mg-2-3X/day topically for 2–3 months	NA	NA	[124]
**Epidermolysis bullosa**							
	CBD	VAS	Double-blind, randomised (*n* = 3)	Topically for 3 months	Faster wound healing Less blistering Pain amelioration	Not reported	[125]

Abbreviations: b.i.d., twice a day; TEWL, Transepidermal water loss; PASI, Psoriasis Area and Severity Index; DLQI, Dermatology Patients’ Quality of Life Index; SCORAD, Scoring atopic dermatitis; POEM, Patient-oriented eczema measure; QOLHEQ, Quality of life hand eczema questionnaire; ISGA, Investigator’s static global assessment; SA-EASI, Self-assessed eczema area and severity index; HRQOL, Health-related quality of life; VAS, Visual Analogue Scale; CBD, Cannabidiol; AD, atopic dermatitis; AE, adverse event; IGA, Investigator Global Assessment; PEA, N-palmitoylethanolamine; AEA, N-acetylethanolamine.

## Data Availability

Not applicable.
